# Using FLO text-messages to enhance health behaviours and self-management of long-term conditions in South-Asian patients

**DOI:** 10.1177/20552076241242558

**Published:** 2024-05-01

**Authors:** Tahreem Chaudhry, Paula Ormandy, Cristina Vasilica

**Affiliations:** School of Health and Society, 7046 University of Salford, Salford, UK

**Keywords:** Mobile health, text messages, long-term conditions, South-Asian, ethnic-minority, self-management, digital health, chronic illness, patient activation

## Abstract

**Objectives:**

Cultural and communication differences faced by South-Asian (SA) ethnic minority groups have led to challenges in the delivery of health care and complex management of long-term conditions (LTCs). We aim to explore the use of text-messaging in SA communities, through the Florence (FLO) health messaging system utilised within U.K. health sectors, to enhance positive health behaviours and self-management.

**Methods:**

A mixed-methods approach was used for this study involving two phases. Phase 1 includes the administration of the patient activation measure to assess SA patient level of skills, knowledge, and confidence to self-manage their LTC; whilst in Phase 2 semi-structured interviews were conducted, exploring the experiences of users and non-users of FLO text messaging to promote self-management behaviours.

**Findings:**

Forty participants (Florence users (n = 20) and non-users (n = 20)) completed the patient activation survey once after using FLO, and took part in interviews. Differences were noted to exist between the two groups (*p* < .01). Users appeared to have higher activation levels and better self-management behaviours (*p* < .01 (*p* = .00). Interviews elicit participants’ perceptions of the text messaging system, along with key themes relative to behavioural constructs: socio-demographical factors; influencing behaviour changes, self-management, and uptake of text messages amongst SA ethnic minorities.

**Conclusion:**

Text messaging and mHealth are being extensively used amongst general populations to monitor and enhance health. The study of SA patient experiences and their use of text messages within the United Kingdom (UK) remains scarce. Therefore, results from the study identify health beliefs that influence patient engagement with digital health interventions and their self-management. Developing policies and culturally appropriate education guidelines for healthcare practitioners can allow for the provision of culturally sensitive interventions tailored in terms of normative, cultural, and religious beliefs; which in turn will address crucial aspects including SA patient information and educational needs supporting their self-management journey.

## Background

South-Asian (SA) populations are four times more likely to develop long-term conditions (LTCs) such as diabetes, chronic kidney disease (CKD) and hypertension compared to Caucasian groups.^1^ There is limited research looking into health-related and self-management behaviours of SA populations. As the SA population in the UK continues to grow it is vital to address the increasing health needs. Indeed, common reasons for non-adherence amongst this population group include health and illness-related beliefs regarding non-adherence; gender roles and cultural priorities; stigma and social support; and communication.^2–5^ These factors are examined in this study, to generate a deeper understanding of how or if they influence self-management behaviours, patient activation and use of technology-based platforms.

Individuals play a large role in determining their outcomes of care and well-being. ‘Patient Activation’ is a behavioural concept defined as ‘*an individual's knowledge, skills and confidence for managing their own health and health care’* (Hibbard JH et al.,^6^ p7). Those with high levels of activation have a better understanding of their role in the care process, with an increased capability of fulfilling that role; and are more likely to engage in positive health behaviours to effectively manage their health and well-being.^7^ Increasing a focus towards the activation levels of those from ethnic minority groups can begin to address the racial disparities and health inequalities, by adopting positive health behaviours and encouraging improved management of LTCs.

The Patient Activation Measure (PAM) has been developed by Hibbard et al.^7^ to capture a patient's beliefs about their ability to self-manage and the likelihood that they would act on those beliefs.^8,9^ The PAM comes in translated versions, including common SA languages. However, there is limited evidence of the application of ‘patient activation’ in SA samples or evidence that these factors are important to trigger behaviours affecting adherence to the treatment regime; which in turn, may affect the activation levels of SA patients.^8^ SAs are reported to face major barriers in the ability to manage their own health and make changes to their lifestyle due to health beliefs, cultural norms, and demographical factors. Assessing patient activation can be useful to gauge SA patient understanding of their condition, and their ability to self-manage. Therefore, the PAM has been used to determine whether it is useful and appropriate, to assess self-management activities and behaviours of SA patients engaging with text messages.

Mobile health (mHealth) technologies are currently transforming the mode and quality of healthcare research on a global scale^9^; particularly in the management of LTCs and preventive health behaviours. One of the simplest and most widely utilised telecommunication systems is SMS or text messaging system via mobile phones, for supporting and monitoring multiple LTCs.^10^ Text messaging platforms can be beneficial by providing information to patients or their carers regarding their condition, by monitoring illness; improving adherence to treatment or medication, or as peer-to-peer networking and support.^10^ Florence (FLO) is a text messaging system developed by NHS Stoke on Trent in 2010, where messages can be adjusted and sent by healthcare providers for each patient depending on their condition. It has been used by over 300,000 people in over 70 health and social care organisations in the United Kingdom and has helped thousands of patients with LTCs such as diabetes and hypertension since 2010.^11^

Evidence verifies the benefits of using text messaging interventions in healthcare; however, much of the focus lies particularly upon European or American populations (ethno-centric samples),^8,12^ compared to a few amongst ethnic groups from SA areas such as India and Pakistan.^13–15^ Although, these few SA studies report such platforms to aid SA patients, by influencing positive behaviours and preventing health-related risks, there is limited evidence suggesting who may or may not use such systems; why it may or may not be used; as well as the behavioural change processes, particularly across SA ethnic-minority groups within the UK. Therefore, to fully understand the uptake of text messages by SA people, it is important to approach participants from the same community or LTC population, who choose not to use or engage with such platforms. Further research is required to understand the cultural barriers and challenges faced by ethnic minority groups living with LTCs, and whether a mobile health platform would be suitable to support their self-management journey. This study aims to address these gaps by exploring the experiences and use of text messages, to enhance health behaviours and self-management in SA patients; by utilising and evaluating the FLO text messaging system, and administering the PAM.

### FLO text messaging system

There is growing interest in the use of the Simple Tele-health NHS Florence system or ‘FLO’, well known within NHS groups, as a tool that can encourage effective clinician-patient interactions to support self-management of patients in the longer term as it is already embedded in practice.^13^ FLO can be used for the management of multiple conditions also, by messages that can be adjusted by healthcare providers for each patient depending on the condition or conditions; defining when messages should be sent; tone of messages; what information they are asking for; and how the system should respond. FLO then sends regular text messages to patients helping them to monitor their health, sharing any information sent back by the patient with the health care provider that is managing their care.

Despite the advancements and rapidly increasing use of text messaging applications, it remains unclear as to whether such systems can be used as an acceptable tool for behaviour change across SAs.^14^ Some studies show such interventions to alter negative behaviours in SA participants, resulting in positive effects on behaviour and improvements in adherence and self-management.^2,3^ In contrast, other studies report a lack of acceptability of mHealth interventions, and non-adherence to treatment regimens in similar samples.^15,16^ Concepts from certain behaviour change theories (Technology acceptance model, Theory of planned behaviour and Health belief model) such as perceived usefulness, persuasion, perceived ease of use, perceptions and attitudes toward technology, motivation, subjective norms and self-efficacy,^17^ have been used to understand and explore adoption and use of technology.^14–16^ Similar determinants have been applied in this study, to understand the acceptance and use of such platforms within an SA sample.

## Methods

### Study site

The study and patient identification took place during NHS clinics that offered FLO as a service, held at the Sandwell and West Birmingham NHS Trust, in the UK. FLO was mainly being used across endocrine and diabetes clinics to monitor important health parameters and to promote self-management of long-term illnesses. Therefore, participants were recruited with conditions such as diabetes, CKD, end-stage renal disease (ESRD) and hyper or hypothyroidism.

### Study phases

**Figure 1.**
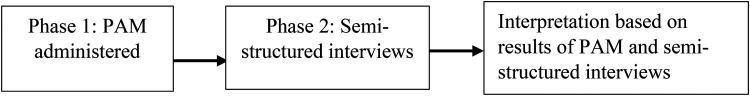
Phases of the study.

The study progressed in two phases (Figure 1). In Phase 1, the patient activation questionnaire was administered once after the use or non-use of FLO. Phase 2 included semi-structured interviews, to explore the experiences of users and non-users of the FLO text messaging system to promote self-management behaviours. Both phases were carried out by the main researcher (TC).

### Phase 1

To address the barriers faced by SAs in regard to self-management, participants completed the PAM to assess and determine their level of knowledge, skills, and confidence; to assume responsibility for their own health, well-being, and self-management. Previous studies outline applications of the tool amongst the general population and identify important patient characteristics that apply to the chosen study sample, that may influence their health and self-management outcomes.^6,8,14^ These include the:

- Ability to self-manage condition, illness, or health problems.
- Ability to engage in activities reducing health declines
- Ability and knowing the importance to intervene in treatment and diagnostic choices
- Ability to collaborate with healthcare providers and organisations
- Ability to navigate health care systems and providers based on performance and quality.

The PAM-13 was used consisting of a five-level Likert scale (*disagree strongly, disagree, agree, agree strongly, N/A*) and 13 items answered according to the patient's level of agreement or disagreement. The short-form 13-item scale was used due to time restrictions with data collection activities and translation of surveys. The scale is a validated and robust tool which has been extensively tested and reviewed by several studies.^15–17^ The PAM has previously been translated and administered in European languages.^13–15^ It is also available in SA languages, however, there is limited evidence of utilising translated versions across ethnic minority groups, particularly SAs. Therefore, the validity of translated PAM surveys was achieved by forward and backward translation^16^; a standardised method commonly used in cross-cultural research.^13–15^ The instrument was translated by SA translators from English to a SA language (Urdu, Gujarati, Hindi, Bengali, Punjabi and Tamil), and back-translated to English, by the researcher with assistance from SA interpreters within the NHS. This was then pilot-tested amongst the translators to assess the time-frame of completion, ensuring it did not interrupt clinic times. Permission was sought from the copyright holder to use the questionnaire prior to administering translated versions.

All questionnaires were self-completed in a language of patient choice. In addition, an indication as to whether the individual was a user or non-user of FLO, was also provided at the start of the survey for the purpose of the study (Appendix 1).

### Phase 2

An inductive approach was used to gather an in-depth understanding of living with an LTC and using text messaging platforms to aid self-management, from a SA participant's perspective. Semi-structured interviews enabled the researcher (TC) to gain rich knowledge and insight about SA individuals, focusing on their experiences, and considering how socio-demographic and cultural factors influence those experiences and behaviour. An interview guide for both users and non-users of the FLO system was designed, pilot-tested, and used for the study (Appendices 2 and 3). This consisted of a set of pre-determined questions to guide the researcher and to follow up on probes to uncover rich data that is individualised, credible and trustworthy. The guide was divided into five concepts and domains to help cover and address health threats, issues related to coping with symptoms, diagnosis, health information and the use of technology.

Concepts of behaviour change theories such as the Common-Sense Self-Regulatory Model (CS-SRM)^13^ and the Health Belief Model (HBM),^17^ were explored and applied to the interviews, to elicit illness-related beliefs perceived by SA populations; probing cultural and religious attitudes that may influence decisions, activation levels and healthy behaviours, related to the acceptance of the FLO text messaging system. The application of the theories was useful amongst the study sample, as the constructs of the models helped to grasp a better understanding of patient health beliefs, cultural norms, practices, and behavioural patterns which in turn predict behaviours regarding the self-management of LTCs.

Interviews followed from the completion of the PAM and lasted between 45 to 60 min. They were audio recorded and transcribed verbatim.

## Ethical approval

Ethical approval was granted by the University of Salford Ethics Committee and the NHS National Research Ethics Service, Health Research Authority (REC Reference: 18/YH/04/36), and the local R & D office. Consent was obtained by the researcher (TC), from all participants who signed informed consent forms regarding their voluntary participation in the study.

## Sample recruitment and consent

Consent was obtained face-to-face from all eligible participants (*n* = 40) recruited from the clinic, to participate in Phases 1 and 2 of the study. The recruitment strategy involved two steps: (1) the lead researcher (TC) distributed information packs to participants, which included the patient information sheet, consent forms and an eligibility criteria checklist, during patient clinical appointments and (2) participants were able to converse and build rapport with the researcher. Questions were answered by the researcher and all the interested participants were asked to complete the eligibility checklist, to determine an appropriate sample selection, prior to consent being obtained. All study subjects completed the PAM followed by interviews within the clinics.

### Participants

#### Inclusion and exclusion criteria

An eligibility checklist (Box 1) was distributed to patients during their clinical appointments prior to obtaining consent. Key patient characteristics included: age, gender, ethnicity, whether patients have an LTC; and whether they were a user or non-user of the FLO text messaging system.

Box 1. Eligibility Inclusion Criteria

- Aged 18 years and above
- Patients managing an LTC
- Patients belonging to a SA population (from countries such as Pakistan, India, Bangladesh, Sri Lanka, and Afghanistan^21^)
- Patients who could provide written informed consent
- Patients already using the ‘FLO’ system
- Patients who have used 'FLO', but may no longer be using it
- Patients who have decided not to use the 'FLO' system

A purposive sampling technique was used to select the most appropriate subjects during clinics, based on the characteristics selected within the study eligibility checklist. Patients who failed to meet the criteria were excluded. Recruitment took place in endocrine clinics where all participants presented with Diabetes. Some individuals had thyroid issues or were multi-morbid with conditions such as such as hypertension, CKD and ESRD.

The aim was to recruit up to the stage where data are saturated and no new themes are emerging; hence, it was deemed appropriate to stop recruitment at forty individuals. Subsequently, it was ensured that an even spread of FLO users, including previous users (*n* = 5), users (*n* = 20), and non-users (*n* = 15) were obtained, along with the number of subjects recruited from certain groups in terms of demographics (gender, age groups, employment, and education levels) to allow for an equal spread of data to be collated. Demographic characteristics of participants are presented in Supplemental Table 1 (Appendix 4).

### Addressing translation and language barriers

Most users of the FLO system were able to speak, read and understand English. However, translators were accessible to those who did not speak English. A few of the non-English speakers were present with a family member who did speak English. All interviews were conducted in a language understood by each research participant, enabling the researcher to communicate effectively. Participants were welcomed to ask a family member to sit through the interview whilst data was collected via questionnaires and interviews, to translate if they did not understand the English language. Alternatively, a translator was provided through language line, a cost-effective and easily accessible service, offering interpreting and translation within healthcare over a simple telephone call. Participants using this system could speak to an individual translating the questions in the same preferred language.

All study instruments such as the PAM, patient information sheets and consent forms were all translated by SA translators from English to a SA language (Urdu, Gujarati, Hindi, Bengali, Punjabi and Tamil), and back-translated to English, by the researcher with assistance from interpreters to increase validity, reliability, effectiveness and efficiency to aid in data collection.^16^

### Obtaining PAM scores

Prior to the analyses, PAM scores were obtained to determine each participant's level of activation (Table 1). Patient responses were converted into PAM scores and then stratified into one of four levels; scores were calibrated based on a scale of 0–100. Permission and a password were needed to access a password-protected software designed by Insignia, which automatically calculated PAM scores using a unique formula. Access was granted by the study site.

**Table 1.** Patient activation levels and cut-off scores for self-management.^21^

Activation level of self-management	PAM scores
One – Low activation	≤47.0
Two – Becoming aware but still struggling	47.1–55.2
Three – Taking action	55.2–67.0
Four – Maintaining behaviours and pushing further	≥ 67.1

## Data analysis

### Statistical analyses (Phase 1)

Challenges were presented in obtaining a license needed for the analyses of the PAM, as the sample was not as large as recommended by Insignia Health (*n* = 200), which is a service that provides the PAM scoring algorithm. Eventually, support was sought through Hibbard et al.^6,7^ and the study site which provided access and assistance with the analyses of smaller samples. Further advice was sought from a statistician on how best to analyse data, and the appropriate tests that could be used for the sample size.

Data collected from the PAM survey was coded and entered in the IBM SPSS 25 statistical package for analysis. Descriptive statistics were conducted for a sub-analysis between demographical variables, use of FLO text messaging and PAM levels. Independent sample *t*-test and one-way ANOVA were carried out, to compare the mean scores within specific subgroups of demographic variables. The findings are discussed below, including responses of FLO users and non-users to the 13-item PAM scale.

## Phase 1 findings

Forty participants were eligible to complete the PAM. Particular demographics were noted in regard to patient activation levels, use or non-use of FLO, and self-management behaviours (discussed below). Participants have been coded as U (user) and NU (non-user), ranging from 1 to 20 as there were twenty in each group (e.g. U1-U20, NU1-NU20).

### Demographics and using FLO

ANOVA was performed to determine whether differences (*p* < .05) existed between users and non-users of FLO, with respect to key variables such as age, gender, ethnicity, religion, educational qualifications or levels, employment status, socio-economic status, and the use of text messaging (or use of mobile phones) to self-manage LTCs (Table 2).

**Table 2.** Mean scores and association between demographic characteristics and use of FLO.

Characteristics	Characteristics associated with use of non-use of FLO	Mean scores (*SD*) FLO users (*n* = 20)	Mean scores (*SD*) FLO non-users (*n* = 20)	Mean scores (*SD*) total sample (*n* = 40)	*df* (total sample)	*F* (Fisher's) value (total sample)	Statistical significance (*p*-value)
Age	Use and non-use of younger participants compared to older participants >18–40 *n* = 14 > 40–60 *n* = 13 > 60 *n* = 13	45.60 (15.80)	57.80 (13.20)	51.70 (15.67)	39	7.0	.01*
Gender	Use and non-use in males and females Males *n* = 19 Females *n* = 21	1.65 (0.49)	1.40 (0.50)	1.50 (0.51)	39	2.54	.12
Ethnicity	Use and non-use in sub-ethnic groups (Indian, Pakistani, and Bengali samples) Indian *n* = 23 Pakistani *n* = 15 Bengali *n* = 2	1.55 (0.51)	1.80 (0.62)	1.50 (0.51)	39	1.96	.17
Education level	Use and non-use in university or high school graduate compared to those not graduated University graduate *n* = 15 Highschool/O levels *n* = 16 Primary level (no graduation) *n* = 9	1.15 (0.37)	1.65 (0.49)	1.93 (0.83)	39	13.40	.001*
Employment status	Use and non-use in those employed and those unemployed or retired Employed *n* = 24 Non-employed *n* = 11 Retired *n* = 5	1.40 (0.50)	1.60 (0.50)	1.50 (0.51)	39	1.58	.00**
Socio-economic status	Use and non-use in those dependent on profession, education levels, finance compared to those who are unemployed and dependent Professional *n* = 15 Admin/secretarial *n* = 5 Machine operative *n* = 4 Retired *n* = 5 Unemployed *n* = 11	2.85 (1.90)	3.20 (1.47)	3.03 (1.69)	39	0.42	.03*
Religion	Use and non-use amongst those who are religious compared to those non-religious Islam *n* = 17 Hinduism *n* = 5 Sikhism *n* = 16 Christianity *n* = 1 No religion *n* = 1	2.15 (1.23)	2.10 (0.97)	2.13 (1.10)	39	0.20	.89

Notes: FLO: Florence; SD, standard deviation; *df*, degree of freedom.

*p < .05, ** *p* < .01.

There was statistical significance found between system use (using or not using the FLO system) and variables such as age (*p* = .01), education levels (*p* = .001) and socio-economic group (*p* = .03). Younger participants between the ages of 25 and 65 years were better activated and adopters to the FLO system, compared to older participants. Differences were found across the study sample in terms of education and literacy levels; which reflected on patient experiences of self-managing their LTCs, and the ability to use the FLO system. All users of the system had some level of education (up to secondary, college or university level), and were able to read messages and communicate their needs. Education appeared to be a major contributor to being able to self-manage and was shown to vary considerably within the SA sub-groups. Ethnicity did not influence FLO use (user *SD* = 1.55, non-user *SD* = 1.80, *p* = .17). However, Indian participants had higher education and activation levels than Pakistani and Bengali participants.

In addition, there was no statistical significance noted in the mean difference between characteristics including gender in terms of users and non-users (user *SD* = 1.40, non-user *SD* = 1.50, *p* = .12); whilst, employment status was significant in mobile phone use (*p* = .00). Most users were employed with a professional background and were adherent to the text messages sent, compared to those who were machine operative or had secretarial roles. Employability also varied between genders, as findings show a majority of SA men to be employed compared to SA women. This has been later explored during interviews.

Health beliefs and religion were also prominent determinants that were noted whilst conversing with participants regarding their LTC, self-management and using FLO. Although literature has cited religion and fatalistic beliefs to be significant in managing illnesses, no statistical significance was detected between religion and using FLO or mobile phone devices (*p* = .89).

### Association between using FLO and patient activation levels

Results showed 70% of participants that were users of FLO to be highly activated at level 4, 95% CI [69.1–78.9], and a *p*-value at *p* < .01 (*p* = .00, 95% CI [74%]), suggesting statistical significance between the two factors.

### Responses to items, patient activation scores and levels of users and non-users

Users appeared to be more highly activated than those not utilising text messages. They expressed an increase in confidence, skills, and ability to independently manage their illness; and text messages are a useful and beneficial platform for appointment reminders, taking medications, positive lifestyle changes and measuring important parameters (blood sugars and blood pressure). In comparison, previous users and non-users had (*n* = 12, 30%) disagreed on certain items based on their understanding of their health problems, and their confidence to consult a healthcare professional when required. The majority of non-users were either categorised in Level 1 (disengaged and overwhelmed stage) or Level 2 (becoming aware but still struggling). 35% of those not using the system were activated at Level 1 with their self-management, leaving only two non-users (10%) who were at Level 4. All users (*n* = 20) appeared to be acting and maintaining healthy behaviours, 85% (*n* = 17) of this group were at Level 4 with their self-management regime. Patients who were at Level 1 or 2, struggled to understand their regimen and how best to manage their LTC independently. Additionally, such participants (55%, *n* = 22) desired more information regarding their illness, of being able to effectively self-manage independently without being reliant on family and relatives.

**Figure 2.**
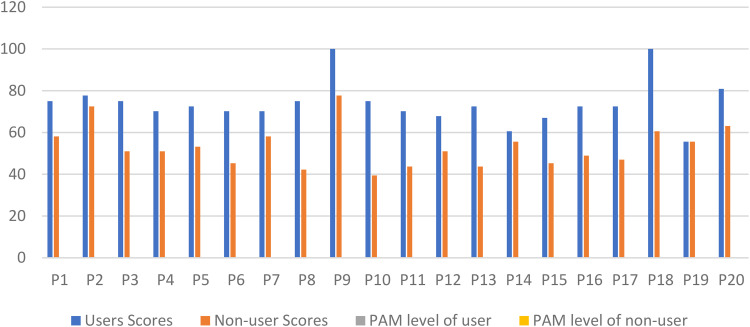
Comparing patient activation scores of users and non-users.

Users and non-users varied considerably as shown in Figure 2, in which scores present a clearer comparison between users and non-users. Items of the survey, patient responses, individual patient activation scores, and levels are also presented in Supplemental Tables 4 and 5 (Appendices 5 and 6), to show further comparisons between the two groups.

## Thematic analyses (Phase 2)

The study utilised a mixed methods approach. Therefore, different analysis techniques were employed to inform the qualitative and quantitative structures. Triangulation was used through the analyses of transcripts and the PAM tool. Data from these were discussed and analysed separately by the research team (TC, PO and CV) to compare, contrast and ensure consensus on themes, and minimising researcher bias. This formulated a relationship between the patient use of FLO text messages and their activation levels (interviews and PAM tool); enabling a deeper understanding of participants’ self-management behaviours and engagement with mobile phone systems. For example, health beliefs in relation to side effects of taking prescribed medications were a recurring theme explored and discussed throughout interviews. This helped explain why some patients were not adhering to their regime and reported having lower patient activation scores.

All interviews (40) were inductively analysed. They were transcribed verbatim and checked for accuracy against audio recording. To identify key themes interviews were read and listened to. Significant words and phrases used by participants were noted, in relation to using or not using text messages and how they influenced behaviours, and were coded alongside a description. The different meanings identified were compared and agreed upon between the lead researcher (TC) and two research team members (PO and CV).

Key themes were identified from the data, with important determinants highlighted and used to theorise and understand self-management behaviours regarding LTCs, and the use of FLO text messages in an SA sample. These include demographics; attitudes and social norms; health and cultural beliefs; coping behaviours, and enhanced self-efficacy and motivation through persuasive messages.

## Phase 2 findings

### Demographics

Certain demographic characteristics related to participant experiences of using and accepting the FLO system, as well as their activation levels including employability, gender role, literacy levels and age. Younger individuals expressed positive experiences compared to older patients:

*I really love using the system for managing my diabetes, however, I do wonder how such a system can be used by someone like my mum, who is in her seventies now, and doesn’t have a clue of how to text. (33-year-old Indian male user, with diabetes and hypertension – U1)*

*I do think the system is helpful as it would display blood results etc. But I don’t feel interested in using technology, maybe it's an age factor. I am too old to take interest in these things now. It is for younger people. (77-year-old Pakistani male non-user, with diabetes, hypertension and CKD – NU2)*

### Attitudes and social norms

Attitudes and social norms influenced FLO use and patient activation levels. Men and women were seen to have distinct roles within the typical SA family system. Women were consumed with responsibilities which included house chores and taking care of the children and the wider extended family. Men were viewed to be the main breadwinners. The majority of non-users were found to be female participants who were housewives with multiple commitments irrelevant to their health, and a lack of interest to use mobile phones (Supplemental Table 6, Appendix 7).

*I don’t really use my phone much … I have too much to do around the house … I am not really interested in texting … I only really use my phone to make important phone calls. Otherwise, I am very busy with household chores and cooking for my husband and family. (53-year-old Indian female non-user, with diabetes, NU3)*

Instead, they expressed the importance of their husband's decisions and involvement regarding their health outcome and whether it was acceptable for them to use FLO. Females who were qualified and employed were more independent, had better health outcomes, engaged with text messages; and were more confident with the prescribed treatment and self-management regime. Such participants emphasised changes in the SA family system and gender equality within the household, where their spouses took responsibility and assisted with cooking and cleaning (Supplemental Table 6, Appendix 7)

Other elements such as social cohesion, connectedness and co-operation were fundamental for SA communities.^19^ Patients were interdependent on relatives, friends, and religious networks as the major source of identity, and protection against the hardships of life such as living with an LTC. During interviews participants identified the important roles of family members and friends in providing information about their condition, accompanying the patient to appointments, helping them use the FLO system, read, or translate text messages; and reminding them to take their medications.

*My sister and myself were shown by the nurse how to administer it for my mum, but then I trained and showed my sister-in-laws how to do it in case I or my sister were not there, so they knew what they are doing now. It's the same with the text messages, we normally read the messages out to her in Bengali to update her on her health. (Daughter of a Bangladeshi female with diabetes assisting mother with the management of diabetes)*

### Health and cultural beliefs

Health-related and cultural beliefs strongly correlated with SA adherence behaviours and participant experiences of self-managing LTCs; affecting engagement with technology and activation levels. Certain subthemes emerged such as the preference for traditional remedies rather than prescribed medications, toxicity of medication, cultural norms, and stigma. Non-adherence and lower patient activation levels were evident amongst SA participants, who expressed their fear of toxicity and believed prescribed medications to have more side effects than actual benefits. SAs who shared such beliefs often turned to alternative remedies and felt they were ‘natural’ and safer. Examples included bitter melon, referred to as ‘Karela’ in some SA languages, and other herbs or vegetables which are a common remedy believed to ‘cure’ diabetes amongst SA samples.

*I do take a lot of bitter melon, my wife always tells me to buy Karela because it is meant to be good for this, some also say to eat pepper especially green pepper, but to eat these vegetables raw. (70-year-old Indian male non-user, with diabetes and hypertension, NU5)*

These remedies were commonly used amongst individuals from the first generation. Use of mobile phones and text messages was not observed amongst this group of participants, due to negative attitudes of them ‘*not working effectively’* or not having the time, or interest to learn how to use a mobile phone. Younger and highly educated individuals from the second or third generation were strictly adherent to their medications and argued there to be limited evidence to suggest the safety or effectiveness of taking traditional remedies. Instead, they expressed that the best way to reduce symptom severity was to adhere to the medications prescribed on time (Supplemental Table 7, Appendix 8).

### Coping behaviours

Interviews explored coping methods adopted by SAs, from which the concept of religion and fatalistic beliefs came about. Commonalities existed amongst Muslim, Hindu, and Sikh participants, with regard to having faith in God. This cultivated positive behaviours, improved well-being, and gave them hope of getting better. Some patients also expressed turning to scriptures, prayer, and counselling with religious clerics to deal with mental distress. All participants were asked about their views of religion and coping, perceptions of God; and self-managing their illness using FLO. The majority of the subjects reported mobile phones and text messages to not intervene with their religiosity and faith in God. Some Muslim and Sikh users found the FLO system beneficial and educational, also stating that God was most ‘*powerful*’, ‘*merciful*’, ‘*benevolent*’, ‘*omniscient’* and *‘omnipresent’;* believing only he can help control symptoms and cure their illnesses. God has been reported to be: incomparable to technology, alleviated feelings of isolation, depression, and anxiety, helped restore faith, cope better, and make sense of their condition. On the other hand, younger subjects had greater belief in medication and scientific evidence, in relation to the causation of an illness rather than God or religion. Such individuals felt more supported through engaging with counsellors, or health care clinicians when support was needed (Supplemental Table 8, Appendix 9).

### Enhanced self-efficacy and motivation through persuasive messages

A strength of using FLO was that it provided persuasive messages, motivated, an increased self-efficacy and activation levels; all assisting SA participants to self-manage their LTC. The majority of FLO users expressed messages to be educational and informative, persuading them to adopt effective lifestyle choices and improve adherence to their prescribed treatment regime. In comparison, many non-users were reluctant to change their lifestyles and explained their lack of motivation was due to not being prompted to look after themselves, or simply not wanting to change (Supplemental Table 9, Appendix 10). A lot of them were unable to engage with the system due to experiencing language barriers, as FLO only delivers messages in the English language. Such individuals made suggestions of how the content of messages can be improved if tailored to be sent at a preferred time, and in a language of their choice:

*I would really like to use FLO; however, I can’t read English. It would be beneficial to design systems that would be more tailored to suit our needs, such as better translated content and services. (67-year-old, Indian female non-user, diabetes and hypertension – U4)*

*I think it would be good if the messages were sent earlier on in the day, to prompt, like I received this message regarding the appointment only yesterday.* *(70-year-old Indian female non-users, unemployed, with diabetes, hypertension and CKD-U5)*

The findings provided new insight into the sequencing of theoretical concepts such as coping, health beliefs, attitudes to health, demographics, motivation, and self-efficacy. Fundamental determinants such as attitudes, subjective norms, health beliefs and perceptions, were also found to contribute to self-management and uptake of text messaging interventions. However, the reasoning behind certain behaviours in relation to inadequate self-management and acceptance of text messaging was not previously explored. Therefore, overlapping constructs of key behaviour change models such as CS-SRM and HBM, have been further explored to depict health behaviours relative to the adoption and acceptance of mHealth interventions in SA populations.

**Figure 3.**
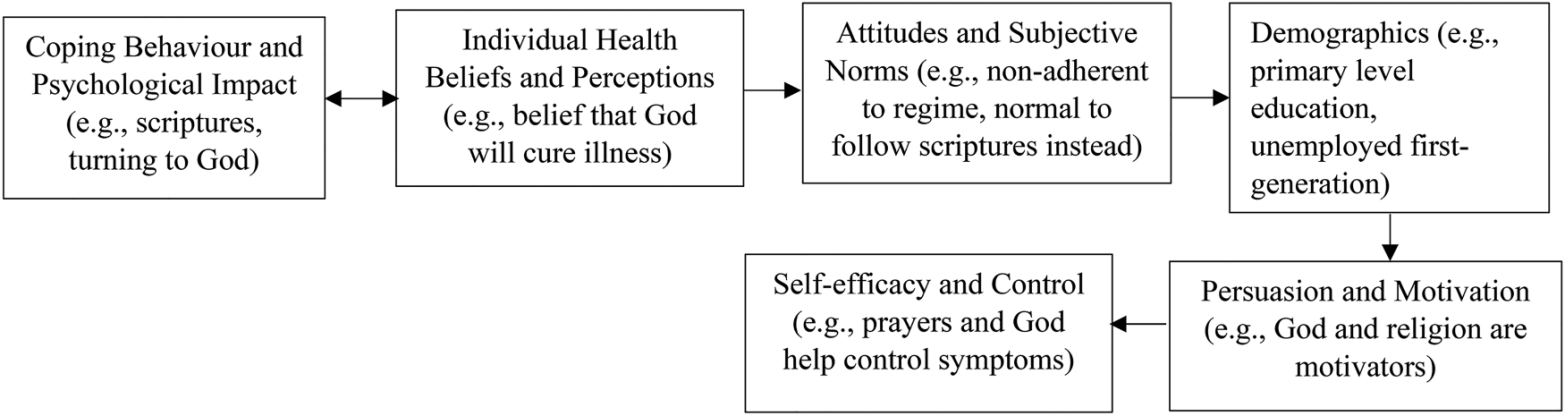
FLO Non-User (NU8) Theoretical Sequence Map of Behaviour Changes and Acceptance of Using Text Messages.

**Figure 4.**
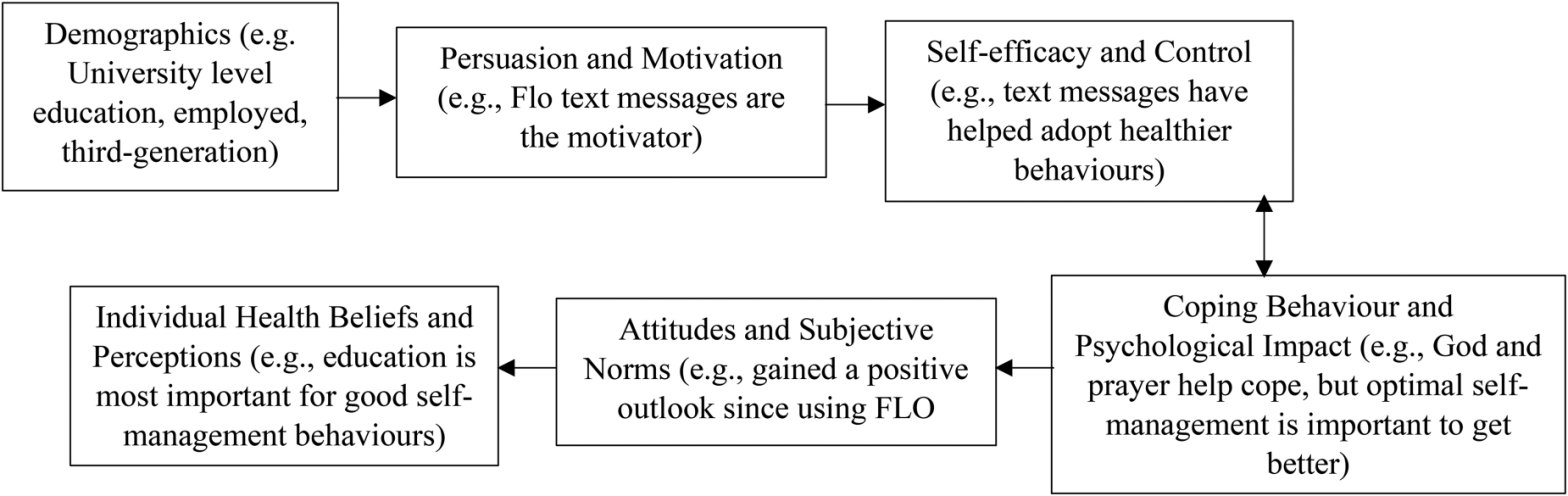
FLO User (U10) Theoretical Sequence Map of Behaviour Changes and Acceptance of Using Text Messages.

The study generated a clearer understanding of how these variables work together and can be applied to understand relevant steps necessary to alter SA behaviours; as well as the acceptance of text messages in certain participants from different contextual backgrounds. The sequencing order of variables and concepts differed for all participants whether users or non-users of FLO, generating individual theoretical sequence maps. For example, for some non-users (NU8) cultural norms were more important than accepting FLO; or belief in God helped them get through it and led them to turn away from FLO. This applied to users (U10), where demographics such as education and employment were seen to contribute to their uptake of technology. Figures 3 and 4 present the same variables depicted in different sequence order dependent on different user and non-user participant contexts (NU8 and U10 refer back to Supplemental Table 8, Appendix 9 for clearer patient context).

FLO: Florence.

FLO: Florence.

## Discussion

The aim of this study was to examine whether the use of text messages and digital health interventions would enhance non-adherence amongst certain SAs, and optimise the self-management of their LTC. The findings support this viewpoint but more significantly provide new depths of knowledge that creates a clearer understanding of the complex barriers faced by SA ethnic minority groups within the UK in regards to their health care needs and outcomes.

The platform has proven to show acceptability amongst this ethnic-minority sample, as individuals mentioned its usefulness and willingness to recommend it to friends and family with similar health issues. On the other hand, FLO was also criticised in terms of language, content, and preferences of other alternative systems. Furthermore, the study contributes to the growing body of evidence surrounding the use of SMS to support LTCs, and particularly research regarding the FLO text messaging platform.^19–21^ However, contextual factors related to those not using the system remain scarce within previous studies^19–21^; hence, addressed and demonstrated in this study through the application of behaviour change theories such as CS-SRM and HBM.

Another fundamental objective of this study was, to assess whether the PAM provides a useful indicator of SA individuals’ ability to self-manage; and whether they would benefit or engage with a text messaging intervention. The tool allowed participants to express their thoughts and perceptions regarding the facilities available to them from the healthcare system, their confidence to consult with their healthcare provider and their understanding of their treatment regime. A valuable factor was that it is available in several different languages including those that were spoken by the participants. The PAM has proven to be a robust and valid instrument, first developed and used in the United States,^6,18,22,23^ and then across European countries,^7,19^ including the UK.^4^ There was notably limited evidence of the tool assessing activation levels in ethnic minority groups. Only two studies were found.^24,25^ Gwyn et al.^24^ assessed activation levels of African-American samples in the United States, and Zeng et al.^24^ across Chinese patients with hypertension and diabetes. The PAM was verified to show good reliability and validity for measuring patient activation levels in both African-American and Chinese participants with LTCs.^24,25^ There were no studies found to employ ethnic minorities in the UK. To address this evidence gap, the 13-item study instrument was useful to initiate discussion and draw out specific patient education, information needs, and the necessity of having culturally tailored educational programmes to enhance their self-management.

Although this study adds to the growing body of evidence, further research is needed to overcome increasing health-related challenges and disparities faced by ethnic minority groups, implementing appropriate mHealth interventions (e.g. FLO) and understand self-management behaviours of SA and similar population groups, particularly since COVID-19. Collaboration between digital health researchers, health care professionals and SA patients may well lead to a greater understanding of what this population desires from an mHealth intervention tailored to their needs to promote effective self-management.^26,27^ This study measured variables (demographics, health beliefs, cultural norms, and religion) in acceptance of FLO and patient activation levels, in a SA population living with LTCs. Interestingly differences were found between age groups and particularly education levels, which helped understand individual preferences and what participants desired from a text-messaging platform to self-manage their LTC. In conclusion, similar study instruments and methods can be used in future to identify the healthcare needs of similar samples. This would aid in developing policies and culturally appropriate education guidelines for healthcare practitioners; to address SA information and educational needs, and allow for the provision of culturally sensitive and tailored digital health interventions, or platforms to support their self-management journey.

## Limitations

Issues with reliability and generalisability may present as a smaller sample was recruited. This led to further issues of obtaining a PAM license for statistical analyses, as a minimum of 200 participants is a requirement. Future studies may seek to employ and administer the PAM across larger ethnic-minority groups in other U.K healthcare settings.

## Implications

This study was conducted employing a robust methodology and theoretical framework to generate findings to understand SAs’ self-management behaviours, their engagement with the FLO system and patient activation levels. Platforms such as FLO were proven to be effective and useful, to aid self-management by providing patient education, motivational messages, and important health reminders. The results contribute to the evidence base on mHealth, and will hopefully endeavour to provide information to improve and guide practice, and generate platforms that will positively benefit the self-management of SA and similar ethnic minority groups.

## Conclusion

Existing evidence relevant to ethnic minorities in the United Kingdom, particularly that of SA patients was found to be extremely depleted. This research provides new depths of knowledge that create a clearer understanding of the complex barriers faced by SA groups within the United Kingdom in regards to their healthcare needs and outcomes. Findings identified health beliefs that influenced patient engagement with digital health (mHealth) interventions and their self-management behaviours, which will help plan and facilitate culturally appropriate systems for SAs and wider ethnic minority populations in the United Kingdom.

## Supplemental Material

sj-docx-1-dhj-10.1177_20552076241242558 - Supplemental material for Using FLO text-messages to enhance health behaviours and self-management of long-term conditions in South-Asian patients Supplemental material, sj-docx-1-dhj-10.1177_20552076241242558 for Using FLO text-messages to enhance health behaviours and self-management of long-term conditions in South-Asian patients by Tahreem Chaudhry, Paula Ormandy and Cristina Vasilica in DIGITAL HEALTH

sj-docx-2-dhj-10.1177_20552076241242558 - Supplemental material for Using FLO text-messages to enhance health behaviours and self-management of long-term conditions in South-Asian patients Supplemental material, sj-docx-2-dhj-10.1177_20552076241242558 for Using FLO text-messages to enhance health behaviours and self-management of long-term conditions in South-Asian patients by Tahreem Chaudhry, Paula Ormandy and Cristina Vasilica in DIGITAL HEALTH

## References

[1] BadawySM BarreraL SinnoMG , et al. Text messaging and mobile phone apps as interventions to improve adherence in adolescents with chronic health conditions: a systematic review. JMIR Mhealth Uhealth 2017; 5: e66.10.2196/mhealth.7798PMC544782528506955

[2] DeSouzaSI RashmiMR VasanthiAP , et al. Mobile phones. The next step towards healthcare delivery in rural India? PLoS ONE 2014; 9: e104895.10.1371/journal.pone.0104895PMC413685825133610

[3] PrinjhaS Ricci-CabelloI NewhouseN , et al. British South Asian patients’ perspectives on a digital health brief messaging system to support medication adherence for type 2 diabetes: a qualitative study (Preprint). JMIR Mhealth Uhealth 2019; 8: 1–13.10.2196/15789PMC719913232310150

[4] Flo | The Health Foundation (2020, November 7th). The Health Foundation. https://www.health.org.uk/news-and-comment/featured-content/power-of-people/flo

[5] GaoJ ArdenM HooZH , et al. Understanding patient activation and adherence to nebuliser treatment in adults with cystic fibrosis: Responses to the UK version of PAM-13 and a think aloud study. BMC Health Serv Res 2019; 19: 1–12.31234848 10.1186/s12913-019-4260-5PMC6591841

[6] HibbardJH GreeneJ BeckerER , et al. Racial/ethnic disparities and consumer activation in health. Health Aff 2008; 27: 1442–1453.10.1377/hlthaff.27.5.144218780935

[7] HibbardJ. (2020, November 5^th^). Supporting people to manage their health: An introduction to patient activation. https://www.kingsfund.org.uk/sites/default/files/field/field_publication_file/supporting-people-manage-health-patient-activation-may14.pdf

[8] McCabePJ Stuart-MullenLG McLeodCJ , et al. Patient activation for self-management is associated with health status in patients with atrial fibrillation. Patient Prefer Adherence 2018; 12: 1907–1916.30288031 10.2147/PPA.S172970PMC6161745

[9] JibeenT . Unconditional self acceptance and self esteem in relation to frustration intolerance beliefs and psychological distress. J of Ration-Emotive & Cognitive-Behavior Therapy 2016; 35: 207–221.

[10] KandulaNR BernardV DaveS , et al. The South Asian healthy lifestyle intervention (SAHELI) trial: protocol for a mixed-methods, hybrid effectiveness implementation trial for reducing cardiovascular risk in South Asians in the United States. Contemp Clin Trials 2020; 92: 105995.32220632 10.1016/j.cct.2020.105995PMC8011000

[11] KrugerD . Psychotherapy research and existential-phenomenological psychology. Duquesne Stud in Phenomenological Psychol 1983; 4: 8–32.

[12] GraffignaG BarelloS BonanomiA , et al. Measuring patient activation in Italy: Translation, adaptation and validation of the Italian version of the patient activation measure 13 (PAM13-I). BMC Med Inform Decis Mak 2015; 15: 1–13.26699852 10.1186/s12911-015-0232-9PMC4690217

[13] KumarK GreenfieldS RazaK , et al. Understanding adherence-related beliefs about medicine amongst patients of South Asian origin with diabetes and cardiovascular disease patients: a qualitative synthesis. BMC Endocr Disord 2016; 16.10.1186/s12902-016-0103-0PMC488088027230479

[14] MohamedAHHM TawfikH Al-JumeilyD , et al. MoHTAM: A Technology Acceptance Model for Mobile Health Applications. 2011 Developments in E-systems Engineering. 2011 Dec;

[15] IslamSM NiessenLW FerrariU , et al. Effects of mobile phone SMS to improve glycemic control among patients with type 2 diabetes in Bangladesh: a prospective, parallel-group, randomized controlled trial. Diabetes Care 2015; 38: e112–e113.10.2337/dc15-050526207059

[16] KamalAK ShaikhQ PashaO , et al. A randomized controlled behavioral intervention trial to improve medication adherence in adult stroke patients with prescription tailored Short Messaging Service (SMS)-SMS4Stroke study. BMC Neurol 2015; 15: 1–13.26486857 10.1186/s12883-015-0471-5PMC4618367

[17] SchepersJ WetzelsM . A meta-analysis of the technology acceptance model: investigating subjective norm and moderation effects. Inf & Manage 2007; 44: 90–103.

[18] HellströmA Kassaye TessmaM FlinkM , et al. Validation of the patient activation measure in patients at discharge from hospitals and at distance from hospital care in Sweden. BMC Public Health 2019; 19: 1–11.31856796 10.1186/s12889-019-8025-1PMC6921492

[19] LinP SimoniJM ZemonV . The health belief model, sexual behaviors, and HIV risk among Taiwanese immigrants. AIDS Educ Prev 2005; 17: 469–483.16255642 10.1521/aeap.2005.17.5.469

[20] LeventhalH PhillipsLA BurnsE . The common-sense model of self-regulation (CSM): a dynamic framework for understanding illness self-management. J Behav Med 2016; 39: 935–946.27515801 10.1007/s10865-016-9782-2

[21] TsangS RoyseC TerkawiA . Guidelines for developing, translating, and validating a questionnaire in perioperative and pain medicine. Saudi J Anaesth 2017; 11: 80.10.4103/sja.SJA_203_17PMC546357028616007

[22] JanzNK BeckerMH . The health belief model: a decade later. Health Educ Q 1984; 11: 1–47.6392204 10.1177/109019818401100101

[23] LucasA MurrayE KinraS. Heath beliefs of UK south asians related to lifestyle diseases: a review of qualitative literature. J Obes 2013; 2013: 1–13.10.1155/2013/827674PMC358818523476751

[24] CundA ConnollyP Birch-JonesJ , et al. Self-management: keeping it simple with FLO*.* Nursing: Research and Reviews 2015; 49(1).

[25] CottrellE CoxT O’ConnellP , et al. Implementation of simple telehealth to manage hypertension in general practice: a service evaluation. BMC Fam Pract 2015; 16: 1–10.26183439 10.1186/s12875-015-0301-2PMC4504444

[26] Penduduk (2020, December 12th). South-Asia sensus. https://sp2010.bps.go.id/index.php/site/tabel?tid=321&wid=0

[27] PooleC. Enabling supported self-management of wound care in a community setting. Nurs Older People 2016; 28: 43–43. Enabling supported self-management of wound care in a community setting. - Abstract - Europe PMC10.7748/nop.28.7.43.s2927573971

