# Supplementary material for: Using FLO text-messages to enhance health behaviours and self-management of long-term conditions in South-Asian patients
Source: Digit Health. 2024 May 1;10:20552076241242558. doi: 10.1177/20552076241242558 (PMC11067678; doi:10.1177/20552076241242558)
Supplement: sj-docx-1-dhj-10.1177_20552076241242558 - Supplemental material for Using FLO text-messages to enhance health behaviours and self-management of long-term conditions in South-Asian patients [file sj-docx-1-dhj-10.1177_20552076241242558.docx]

**COREQ (COnsolidated criteria for REporting Qualitative research) Checklist**

A checklist of items that should be included in reports of qualitative research. You must report the page number in your manuscript or provide a description of where you consider each of the items listed in this checklist. If you have not included this information, either revise your manuscript accordingly before submitting or note N/A

| Topic | | Item No. | Guide/Description | Description/page number |
| --- | --- | --- | --- | --- |
| **Domain 1: Research Team and reflexivity** | |  |  |  |
| *Personal characteristics* | |  |  |  |
| Interviewer/facilitator | | 1 | Which author/s conducted the interview or focus group? | Pg6 (TC) |
| Credentials | | 2 | What were the researcher’s credentials? E.g. PhD, MD | Title page, PhD |
| Occupation | | 3 | What was their occupation at the time of the study? | Pg6 (Researcher stated) |
| Gender | | 4 | Was the researcher male or female? | Female |
| Experience and training | | 5 | What experience or training did the researcher have? | PhD (credentials and level of experience reported on title page) |
| *Relationship with participants* |  | |  |  |
| Relationship established | | 6 | Was a relationship established prior to study commencement? | Reported on pg7 |
| Participant knowledge of the interviewer | | 7 | What did the participants know about the researcher? e.g. personal goals, reasons for doing the research | Participants were all informed the reasons were doing the research which was included in the PIS (reported on pg7) |
| Interviewer characteristics | | 8 | What characteristics were reported about the inter viewer/facilitator? e.g. Bias, assumptions, reasons and interests in the research topic | Interests in the research topic- reported on pg6 (Phase two section) |
| **Domain 2: Study Design** | |  |  |  |
| *Theoretical Framework* | |  |  |  |
| Methodological orientation and Theory | | 9 | What methodological orientation was stated to underpin the study? e.g. grounded theory, discourse analysis, ethnography, phenomenology, content analysis | Content analysis or analyses of the data collected discussed in data analyses-pg16 |
| *Participant Selection* | |  |  |  |
| Sampling | | 10 | How were participants selected? e.g. purposive, convenience, consecutive, snowball | Purposive sampling-reported pg8 |
| Method of approach | | 11 | How were participants approached? e.g. face-to-face, telephone, mail, email | Reported on pg7- Sample and Recruitment section |
| Sample size | | 12 | How many participants were in the study? | Reported on Pg2 and pg7 |
| Non-participation | | 13 | How many people refused to participate or dropped out? Reasons? | 0-NA |
| *Setting* | |  |  |  |
| Setting of data collection | | 14 | Where was the data collected? e.g. home, clinic, workplace | Clinics- reported on pg7 |
| Presence of nonparticipants | | 15 | Was anyone else present besides the participants and researchers? | Family members of participants attending clinics- Reported on pg 8 |
| Description of sample | | 16 | What are the important characteristics of the sample? e.g. demographic data, date | And data reported on pg9 and pg16-19 |
| *Data collection* | |  |  |  |
| Interview Guide | | 17 | Were questions, prompts, guides provided by the authors? Was it pilot tested? | Reported on pg6 |
| Repeat interviews | | 18 | Were repeat inter views carried out? If yes, how many? | NA |
| Audio/visual recording | | 19 | Did the research use audio or visual recording to collect the data? | Audio- Reported on pg 7 |
| Field notes | | 20 | Were field notes made during and/or after the interview or focus group? | NA |
| Duration | | 21 | What was the duration of the interviews or focus group? | 45-60minutes-Reported on pg7 |
| Data saturation | | 22 | Was data saturation discussed? | Yes-reported on pg8 |
| Transcripts returned | | 23 | Were transcripts returned to participants for comments or corrections? | No-NA |
| **Domain 3: Analysis and findings** | |  |  |  |
| *Data analysis* | |  |  |  |
| Number of Data Coders | | 24 | How many data coders coded the data? | 3- reported on pg16 |
| Description of the coding tree | | 25 | Did authors provide a description of the coding tree? | Described as key themes- Reported and relative to themes on pg16 |
| Derivation of themes | | 26 | Were themes identified in advance or derived from the data? | Derived from data, reported on pg16 |
| Software | | 27 | What software, if applicable, was used to manage the data? | NA- non used for this research hand-transcribed by research team |
| Participant checking | | 28 | Did participants provide feedback on the findings? | NA |
| *Reporting* | |  |  |  |
| Quotations presented | | 29 | Were participant quotations presented to illustrate the themes/findings? Was each quotation identified? e.g. participant number | Reported pg17-19 and pg34-40 |
| Data and findings consistent | | 30 | Was there consistency between the data presented and the findings? | Reported on triangulation and findings on p16 |
| Clarity of major themes | | 31 | Were major themes clearly presented in the findings? | Reported pg17-19 |
| Clarity of minor themes | | 32 | Is there a description of diverse cases or discussion of minor themes? | Reported in phase two findings- pg16-19 |
