# Supplementary material for: Using FLO text-messages to enhance health behaviours and self-management of long-term conditions in South-Asian patients
Source: Digit Health. 2024 May 1;10:20552076241242558. doi: 10.1177/20552076241242558 (PMC11067678; doi:10.1177/20552076241242558)
Supplement: sj-docx-2-dhj-10.1177_20552076241242558 - Supplemental material for Using FLO text-messages to enhance health behaviours and self-management of long-term conditions in South-Asian patients [file sj-docx-2-dhj-10.1177_20552076241242558.docx]

**Supplementary Material**

## **Appendix 1.** *Patient Activation Measure Used for Study*

*Are you a user of the FLO text messaging system? Please circle the answer that applies*

*Yes No*

**1= Disagree Strongly 2= Disagree, 3=Agree, 4= Agree Strongly 5 N/A**

|  | *Statement* | 1 | 2 | 3 | 4 | 5 |
| --- | --- | --- | --- | --- | --- | --- |
| 1 | When all is said and done, I am the person who is responsible for take care of my health | 1 | 2 | 3 | 4 | 5 |
| 2 | Taking an active role in my own health care which is the most important thing that affects my health | 1 | 2 | 3 | 4 | 5 |
| 3 | I am confident I can help prevent or reduce problems associated with my health | 1 | 2 | 3 | 4 | 5 |
| 4 | I know what each of my prescribed medications do | 1 | 2 | 3 | 4 | 5 |
| 5 | I am confident that I can tell whether I need to go to the doctor or whether I can take care of a health problem myself | 1 | 2 | 3 | 4 | 5 |
| 6 | I am confident that I can tell a doctor my concerns I have even when he or she does not ask | 1 | 2 | 3 | 4 | 5 |
| 7 | I am confident that I can follow through on medical treatments I may need to do at home | 1 | 2 | 3 | 4 | 5 |
| 8 | I understand my health problems and what causes them | 1 | 2 | 3 | 4 | 5 |
| 9 | I know what treatments are available for my health problems | 1 | 2 | 3 | 4 | 5 |
| 10 | I have been able to maintain (keep up with) lifestyle changes, like eating right or exercising | 1 | 2 | 3 | 4 | 5 |
| 11 | I know how to prevent problems with my health | 1 | 2 | 3 | 4 | 5 |
| 12 | I am confident I can figure out solutions when new problems arise with my health | 1 | 2 | 3 | 4 | 5 |
| 13 | I am confident that I can maintain lifestyle changes, like eating right and exercising, even during times of stress | 1 | 2 | 3 | 4 | 5 |

**Appendix 2.** *Interview guide for Users of FLO text messaging System*

| **Concepts and Domains** | **Discussion** |
| --- | --- |
| Personal and socio-demographic characteristics | Factors such as education level and gender, and how this affects their adherence regime and whether these factors affect their views of intervention |
| Technology | For how long have you been using FLO?  How do you find the system?  Have you experienced any challenges with the system?  What will you improve/change? |
| Self-management | Are your symptoms of the disease well-controlled?  How do you feel about your condition?  Do you feel supported, are there any emotional consequences such as depression, anxiety?  Do you understand the actual cause of your illness? the number of symptoms you see as part of your illness and experiences of it, whether text messages provided any support of identifying symptoms of certain conditions  What do you understand about your treatment regime?  Do you feel that medications help, or are you dependent on alternative therapies?  Has the text messaging intervention help prompt you to take medications on time, exercising frequently and/or promoting healthy eating?  Do you feel supported from text messages?  Do the messages provide relevant information required to manage disease effectively?  Do text-messages prompt you regarding when to visit your health care providers?  Do you face difficulties/barriers with the medication regime/self-management regime? |
| Health beliefs and Behavioural outcomes | Is the intervention beneficial to your health?  Do you have any negative views towards the text messaging system?  Do you feel that text messages help changes behaviour in positive ways or negative way?  Do the messages promote healthy behaviours e.g. healthy eating, exercising?  How interested do you feel about your condition and the management?  Do text messages make you feel motivated about their management?  Do you have any fears about the consequences of their condition?  Do you show poor self-management measures? If so why? |
| Religious and fatalistic factors | Does religion play a part in the management of your condition?  Do you feel that a ‘higher power’ helps to deal with the condition rather than medications?  Do you feel that god helps get through it?  In general, would you say that your health condition results from god and believe that god is the only cure?  Does text messaging intervene with god and religion?  Do you feel that your faith is a stronghold even during hard times they face in relation to your disease?  Do cultural traditions and customs play a part in self-management? (such as different roles/responsibilities between men and women in terms of cultural norms)  Do you prefer traditional medications rather than ‘western medications? |

**Appendix 3.** *Interview guide for Non-users of FLO text messaging System*

| **Concepts and Domains** | **Discussion** |
| --- | --- |
| Personal and socio-demographic characteristics | Factors such as education level and gender, and how this affects their adherence regime and whether these factors affect their views of intervention |
| Technology | Have you used FLO or any other text messaging intervention in the past?  If you have,   - What was your experience of using the system? - Have you experienced any barriers and challenges? - Why have you decided not to use anymore?   If you haven’t   - Why you have not engaged with the technology? - What were the key challenges?   How do you envisage a text intervention to be to meet your needs? |
| Self-management | Are your symptoms of the disease well-controlled?  How do you feel about your condition?  Do you feel supported, are there any emotional consequences such as depression, anxiety?  Do you understand the actual cause of your illness? the number of symptoms you see as part of your illness and experiences of it  Do you feel that you would be better supported if using interventions/ systems such as text messages of identifying symptoms of certain conditions?  What do you understand about your treatment regime?  Do you feel that medications help, or are you dependent on alternative therapies?  Are you to taking medications on time, exercising frequently and/or promoting healthy eating?  Do you feel supported from your health care provider?  Do you have the relevant information required to manage disease effectively from your health care providers?  Do you visit your health care providers, when required?  Do you face difficulties/barriers with the medication regime/self-management regime?  Do the messages provide relevant information required to manage disease effectively?  Do you visit your health care providers, when required? |
| Health beliefs and Behavioural outcomes | Does your current treatment regimen show to have any benefits to your health outcomes?  How interested do you feel about your condition and the management?  Do you feel that a text messaging intervention would aid in self-management if you were to engage with such systems?  Do you feel that using a text-messaging intervention will have a positive or a negative impact on health behaviour outcomes?  Do you have any fears about the consequences of condition?  Do you show poor self-management measures? If so why? |
| Religious and fatalistic factors | Does religion play a part in the management of your condition?  Do you feel that a ‘higher power’ helps to deal with the condition rather than medications?  Do you feel that god helps get through it?  In general, would you say that your health condition results from god and believe that god is the only cure?  Do you feel that a text messaging intervention intervene with god and religion? Is this your reason for not engaging within such interventions?  Do you feel that your faith is a stronghold even during hard times they face in relation to your disease?  Do cultural traditions and customs play a part in self-management? (such as different roles/responsibilities between men and women in terms of cultural norms)  Do you prefer traditional medications rather than ‘western medications? |

**Appendix 4: Table 1.** *Demographic Characteristics of Participants*

| **Gender** | **FLO users/non-users** | **Age Groups** | **Religion/Faith** |
| --- | --- | --- | --- |
| Male n= 19 (47.5%)  Female n= 21 (52.5%) | FLO Users= 20 (50%)  FLO Non-users= 20 (50%) | 18<40 n= 13 (32.5%)  40<60 n= 14 (35%)  >60 n= 13 (32.5%) | Islam n=17 (42.5%)  Hinduism n= 5 (12.5%)  Sikhism n= 16 (40%)  Christianity n= 1 (2.5%)  No religion=1 (2.5%) |
| **Modality Group** | **Ethnic Group** | **Socio-economic Group** | **Employment Status** |
| Diabetes n= 16 (40%)  Hypertension n=16 (40%)  CKD/ESRD n= 5 (12.5%)  Thyroid n= 3 (7.5%) | Indian n= 23 (57.5%)  Pakistani n= 15 (37.5%)  Bengali n= 2 (5%) | Professional n= 14 (35%)  Administrative/  Secretarial n= 2 (5%)  Machine operatives= 4 (10%)  Unemployed or retired n= 20 (50%) | Employed= 20 (50%)  Unemployed= 20 (50%) |

**Appendix 5: Table 4.** *PAM Survey Items and Patient Responses*

| **Item from PAM** | **Level of Dis/Agreeability** | **Users (including previous users) (n=20)** | **Non-Users (n=20)** |
| --- | --- | --- | --- |
| When all is said and done, I am the person who is responsible for taking care of my health | *Agree Strongly*  *Agree*  *Disagree Strongly*  *Disagree*  *N/A* | 20 (100%)  0 (19%)  0  0  0 | 5 (25%)  10 (50%)  0  5 (25%)  0 |
| Taking an active role in my own health care which is the most important thing that affects my health | *Agree Strongly*  *Agree*  *Disagree Strongly*  *Disagree*  *N/A* | 15 (75%)  5 (25%)  0  0 | 8 (40%)  9 (45%)  0  3 (15%) |
| I am confident I can help prevent or reduce problems associated with my health | *Agree Strongly*  *Agree*  *Disagree Strongly*  *Disagree*  *N/A* | 12 (60%)  7 (35%)  0  1 (5%)  0 | 6 (12.3%)  8 (40%)  6 (13.3%)  0  0 |
| I know what each of my prescribed medications do | *Agree Strongly*  *Agree*  *Disagree Strongly*  *Disagree*  *N/A* | 16 (80%)  4 (20%)  0  0  0 | 5 (25%)  8 (40%)  0  7 (35%)  0 |
| I am confident that I can tell whether I need to go to the doctor or whether I can take care of a health problem myself | *Agree Strongly*  *Agree*  *Disagree Strongly*  *Disagree*  *N/A* | 10 (50%)  8 (40%)  0  2 (10%)  0 | 3 (15%)  7 (35%)  0  10 (50%)  0 |
| I am confident that I can tell a doctor my concerns I have even when he or she does not ask | *Agree Strongly*  *Agree*  *Disagree Strongly*  *Disagree*  *N/A* | 17 (85%)  2 (10%)  0  1 (5%)  0 | 6 (30%)  5 (25%)  0  9 (45%)  0 |
| I am confident that I can follow through on medical treatments I may need to do at home | *Agree Strongly*  *Agree*  *Disagree Strongly*  *Disagree*  *N/A* | 12 (60%)  8 (40%)  0  0  0 | 0  7 (35%)  11 (55%)  2 (10%) |
| I understand my health problems and what causes them | *Agree Strongly*  *Agree*  *Disagree Strongly*  *Disagree*  *N/A* | 14 (31%)  6 (25%)  0  0  0 | 0  8 (40%)  0  12 (60%)  0 |
| I know what treatments are available for my health problems | *Agree Strongly*  *Agree*  *Disagree Strongly*  *Disagree*  *N/A* | 9 (45%)  11 (55%)  0  0  0 | 4 (20%)  9 (45%)  0  7 (35%)  0 |
| I have been able to maintain (keep up with) lifestyle changes, like eating right or exercising | *Agree Strongly*  *Agree*  *Disagree Strongly*  *Disagree*  *N/A* | 12 (60%)  8 (40%)  0  0  0 | 4 (20%)  8 (40%)  5 (25%)  3 (15%)  0 |
| I know how to prevent problems with my health | *Agree Strongly*  *Agree*  *Disagree Strongly*  *Disagree*  *N/A* | 6 (30%)  14 (70%)  0  0  0 | 0  10 (50%)  4 (20%)  6 (30%)  0 |
| I am confident I can figure out solutions when new problems arise with my health | *Agree Strongly*  *Agree*  *Disagree Strongly*  *Disagree*  *N/A* | 8 (40%)  11 (55%)  0  1 (5%)  0 | 0  6 (30%)  8 (40%)  6 (30%)  0 |
| I am confident that I can maintain lifestyle changes, like eating right and exercising, even during times of stress | *Agree Strongly*  *Agree*  *Disagree Strongly*  *Disagree*  *N/A* | 8 (40%)  12 (60%)  0  0  0 | 0  9 (45%)  0  11 (55%)  0 |

## **Appendix 6: Table 5.** *PAM scores and Levels of Users and Non-users*

| **Patient ID** | U/NU1 | U/NU2 | U/NU3 | U/NU4 | U/NU | U/NU6 | U/NU | U/NU8 | U/NU9 | U/NU10 | U/NU11 | U/NU12 | U/NU13 | U/NU14 | U/NU15 | U/NU16 | U/NU17 | U/NU18 | U/NU19 | U/NU20 |
| --- | --- | --- | --- | --- | --- | --- | --- | --- | --- | --- | --- | --- | --- | --- | --- | --- | --- | --- | --- | --- |
| **Users Scores** | 75.00 | 77.70 | 75.00 | 70.20 | 72.50 | 70.20 | 70.20 | 75.00 | 100.00 | 75.00 | 70.20 | 67.80 | 72.50 | 60.60 | 67.00 | 72.50 | 72.50 | 100.00 | 55.60 | 80.90 |
| **Non-user Scores** | 58.10 | 72.50 | 51.00 | 51.00 | 53.20 | 45.30 | 58.10 | 42.20 | 77.70 | 39.40 | 43.70 | 51.00 | 43.70 | 55.60 | 45.30 | 48.90 | 47.00 | 60.60 | 55.60 | 63.10 |
| **PAM level of user** | Level 4 | Level 4 | Level 4 | Level 4 | Level 4 | Level 4 | Level 4 | Level 4 | Level 4 | Level 4 | Level 4 | Level 3 | Level 4 | Level 3 | Level 4 | Level 4 | Level 4 | Level 4 | Level 3 | Level 4 |
| **PAM level of non-user** | Level 3 | Level 4 | Level 2 | Level 2 | Level 2 | Level 1 | Level 3 | Level 1 | Level 4 | Level 1 | Level 1 | Level 2 | Level 1 | Level 3 | Level 1 | Level 2 | Level 1 | Level 3 | Level 3 | Level 3 |

##

## **Appendix 7: Table 6.** *Familial Roles Influencing Acceptance and Use of Text Messages*

| **U3- Context-** Female (37), Indian, attended secondary school in India, unemployed, newly diagnosed with diabetes. Hindi speaker, does not speak English very well. | **NU3- Context-** Female (42), Indian, attended college, employed. Type 1 diabetes for 24 years and hypertension developed recently. She expresses her views of education being an important factor when learning to self-manage a LTC, and FLO assisting her to do better. |
| --- | --- |
| **FLO Non-user** | **FLO User** |
| [General views of FLO*]: “I don’t find the system interesting to use, My English is not great I don’t think I will be able to adhere to messages very well.”*  [Familial roles/adoption to FLO]: “*I don’t really use my phone much… I have too much to do around the house… I am not really interested in texting…I only really use my phone to make important phone calls. Otherwise, I am very busy with household chores and cooking for my husband and family.”* | [General views of FLO]: *“I think systems such as FLO are brilliant, especially to help remind me to check my parameters at the right times. The messages are educational with advice of controlling my blood sugars.”*  [Familial roles/adoption to FLO]: *“I am able to engage with text messages and run my household. I get a lot of help from my husband and kids. Since I have signed up to it and started changing my lifestyle, they have joined me.”* |
| Level 1 - Low activation level, less concerned with taking responsibility for self-management. | Level 4 - High Patient activation levels and increased engagement with FLO text messages. |
| *“I don’t really make my own decisions, my husband has supported me since I have come from India, he attends my appointments and helps me make the best choice for us.”*  *“I don’t think he would be too happy for me to engage with a system I am not too familiar with, plus I have many responsibilities at home.”* | *“You have to make time for your appointments, and just your well-being as a whole. Education is very important especially when trying to understand your illness or condition. I have the knowledge to self-manage my condition well.”*  *“Ever since FLO I am always learning something new, and my family are very supportive and want to learn with me so that they can help in case of a hypo, or anything else that can go wrong.”* |

## **Appendix 8: Table 7.** *SA Health Beliefs, Perceptions, and Acceptance to Text Messages*

| **NU6 Context-** *Fear of Side-effects: 1^st^ generation*  Female (69), Indian, has primary education in India, unemployed. Diabetes, hypertension and CKD. Feeling quite anxious and scared for taking multiple medications to treat her symptoms, which may increase risk of side-effects | **NU7 Context -** *Trial and use of herbal/ alternative remedies:1^st^ generation*  Male (70), Indian, secondary school in India, retired. Diabetes and hypertension. Experiences of trying herbal remedies as an alternative to prescribed medications. Mentions the benefits of them being natural and *“good for you”* | **U6 Context -** *Trial and use of herbal/alternative* *remedies: 1^st^ generation*  Female (58), Pakistani, university in Pakistan. Diabetes, hypertension and CKD. FLO had positive outcome on her health. Receives support from her daughters with messages as not great with technology. Patient can communicate and read in English. Visited a *“Hakim”* (herbalist/ or allopathic practitioner) in Pakistan who informed her that diabetes could be cured through herbal medications he gave to her. | **U7 Context -** *Trial and use of herbal/alternative remedies: 2^nd^ generation*  Female (44), Indian, secondary level (college) education, employed. Attending clinics due to her type 2 diabetes (has developed hypertension). Reported improved symptom control and positive outlook after using FLO. Speaks about her experiences of going to India to visit relatives who advised her to try herbal remedies as a cure to diabetes. | **U8 Context -** *Experiences of stigma attached to condition: 3^rd^ generation*  Female (33), Indian, secondary level (college) education, employed. Attending follow-up clinic for her diabetes (has developed hypertension). Prefers the FLO system in comparison to ‘Patient Knows’. Discusses the stigmas that exist in her family with regards to diabetes treatments such as insulin administration. |
| --- | --- | --- | --- | --- |
| **FLO Non-user** | **FLO Non-user** | **FLO User** | **FLO User** | **FLO User** |
| [General views/perception of FLO]: *“Generally, from the information you have provided about the system, it sounds pretty good, especially for those needing reminding to check blood sugars and blood pressure daily.”*  [Health beliefs/adoption of FLO]: *“I am not saying that the system is not good, it’s just that I don’t think using it will reduce the number of tablets I am taking it will only remind me to take all of them, which is what I am trying to avoid. I believe taking too many medications are bad for my health, however, doctors have told me the opposite that they are there to help me. If they want me to use the system [referring to FLO], they need to understand my beliefs."* | [General views/perception of FLO]: *“I am not really a technology user; throughout my life I have not found it important to use it. I am quite old-fashioned and much rather prefer to receive information and updates via the post.”*  [Health beliefs/adoption of FLO]: *“I don’t think I could keep up with FLO. It wouldn’t help me; I am not used to the mobile phone as I have never been interested in learning how to use it. I also don’t believe that texts will cure my disease. I am happy with the remedies I am trying such as Karela (Bitter melon) juice, which I think are more effective than English [referring to prescribed medications] medicine.”* | [General views/perception of FLO]: “*FLO has helped me; the texts are very good. I manage with the system but sometimes when I need to send my readings for blood sugars or blood pressure, I ask my daughter to help me. I think translated messages would be better.”*  [Health beliefs/adoption of FLO]: *“I think my views regarding FLO and my illnesses are separate. I do believe the system to have helped me personally with my conditions. However, I also feel bad for wasting my money and believing that the remedy given to me by the herbalist can cure me. I have learnt that there is no cure to the diabetes…The system has improved my self-management. Therefore, I will continue to use it.”* | [General views/perception of FLO]: *“If it was* *not for FLO, I think I would be forgetting to take my medications on time and have difficulties to remember to monitor my blood sugars. I have a busy schedule so having FLO is convenient.”*  [Health beliefs/adoption of FLO]: *“I have tired herbal remedies when I went to India…I will never go back to using them again. I think the most beneficial thing is my routine, which is to take the medications my health care provider has told me to. Ever since my doctor has signed me up to FLO, I have gained more confidence in managing my diabetes.”* | [General views/perception of FLO]:*” I really love FLO I have been using ‘Patient Knows’ but that only shows me my blood test results. On the other hand, there’s FLO which has really helped me keep on track with my diabetes care. I love receiving reminder texts regarding my blood sugars and updates on my treatment options. “*  *“FLO is quicker and easier to use and messages are quick to access; whereas, when using ‘Patient Knows’ I normally have to login and wait for it to upload, and it can sometimes take a while*.”  [Health beliefs/adoption of FLO]: *“There are certain health beliefs in the SA culture, especially a stigma for things like cancer and diabetes. There are a few people with diabetes in my family and they all tend to keep insulin administration a secret, I think it’s because they find it embarrassing and wouldn’t want the wider SA community finding out.”*  *“Although, there are health beliefs in our culture regarding illnesses such as diabetes, I never let it get in the way of my regime or even my use of FLO. It’s quite irrelevant to me, I only care about my health and that’s it.”* |
| Level 1- Poor self-management and low activation levels as reluctant to take medications, due to health beliefs regarding side-effects. | Level 1 - Poor self-management and low activation levels, due to health beliefs regarding alternative therapies. Does not fully understand the full benefits of taking prescribed medications. | Level 3 - Good self-management regime, following prescribed regime. Stopped the herbal remedies and adherent to messages sent. High activation level, has a good understanding of her treatment regime. | Level 4 - Good self-management, following prescribed regime. Finds messages useful, increased confidence and activation levels. | Level 4 - Good self-management, following prescribed regime. Finds messages useful, increased confidence and activation levels. |
| *“I take eight tablets in the morning and nine in the afternoon, I asked him [referring to doctor] to reduce the number of tablets due to the side-effects associated with them, but the doctors said no.”*  *“Even my GP was very surprised too. I was beginning to think I will develop a lot of side effects. I stopped them all because of that, I now only take metformin.”* | *“I do take a lot of bitter melon, my wife always tells me to buy Karela because it is meant to be good for this, some also say to eat pepper especially green pepper, but to eat these vegetables raw.”*  *“I don’t think a mobile phone system would be satisfying for me because I think it will just be sending me instructions rather than have an understanding of my needs. I think these remedies are working for me because they go through my blood stream, it may even cure me.”* | *“I went to Faisalabad (city in Pakistan) to see a hakim and they told me that they could cure my diabetes.”*  *“I really should not have been so naive; I am going to continue to stick to what my doctor and the FLO system advise me to do.”* | *“I have eaten bitter melon as well, I have also drunk water of that, I have had people from our community show me videos that if you dip your feet in crushed karela that it will work, I was like that’s not going to work… it’s got to get into your blood. I guess it can help thin the blood, as it is very healthy, it is my favourite vegetables but that does not mean it will cure my diabetes, it won’t replace my insulin, I wish it would but it’s just another alternative to you know…but in reality, it won’t control your blood sugars.”*  *“It’s a proven fact, for example, If I stopped taking my insulin I would have fallen and I’d be admitted. The herbal medications are not proven to be scientifically effective, they’re not real medications. If they were proven to be good for you, I would take them.”* | *“I am not sure what the big issue is, especially with injecting insulin in public. The grown-ups in my family [referring to first generation family members] need to be better educated in regards to their illness. There needs to be a less stigmatised view and better self-management. I think a system like FLO can really help people like my mum.”* |

## **Appendix 9: Table 8.** *SA Participants View on Coping with their LTC*

| **NU7 Context**  Female (60), Pakistani Muslim, primary school in Pakistan, unemployed. Diabetes and hypertension. Practising Muslim - God and religion give her hope help her think positively about her illness. Non-user, using FLO would not give her hope the same way God and religion does. | **NU8 Context**  Male (58), Pakistani Muslim, college, employed.  Diabetes and hypertension, secondary CKD, a year ago. Believes in predestination and that the onset of his LTC was meant to be - God will, He is the only one that can make him better. For these reasons he does not adhere too his medications, and not interested in using FLO to optimise self-management. | **NU9 Context**  Female (38), Indian Hindu, university, employed. Diabetes less than a year ago, experiencing depression. Does not practice religion. Relies on friends for emotional support, seeing a counsellor/healthcare provider when anxious about her condition. Feels FLO would help with her self-management especially taking medications on time. | **U9 Context**  Female (55) Pakistani Muslim, college in Pakistan, unemployed. Diabetes and hypertension two years ago. Her faith in God, helps her to cope since her diagnosis. Finds FLO very helpful with regard to her self-management. | **U10 Context**  Female (35), Indian Hindu, university, employed. Diabetes a few months ago, also depressive symptoms. Prayer and going to the temple makes her feel happier. Since using FLO, she has gained a more positive outlook on her self-management. Being educated plays a key role in her ability to self-manage. |
| --- | --- | --- | --- | --- |
| **FLO Non-user** | **FLO Non-User** | **FLO Non-User** | **FLO User** | **FLO User** |
| [General views/ perception of FLO]: *“I am not a FLO user but I think it can assist and improve self-management for those struggling. I am doing fine for now so I don’t think I need to use it.”*  [Coping, Religious views/ and adoption to FLO]: *“God is enough for me, He helps me get through it. I don’t need to use FLO. If I have God, I don’t need anything else.”* | [General views/perception of FLO]: “*I am not too keen to use FLO, I don’t have interest in mobile phones or texting. I barely use my phone I am busy with work.”*  [Coping, Religious views/ adoption to FLO]: *“If God has predetermined my illness, then only he will make it better, I don’t believe in using a text messaging system to try improve my health. Only God can make me improve.”* | [General views/perception of FLO]: *“I think a system like FLO would really help me remember to take my medications on time and check my blood sugars when needed.”*  [Coping, Religious views/adoption to FLO]: *“I don’t really think religion or God has any relevance to my condition. Although, I think FLO would help me improve my adherence, I really struggle with taking my tablets on time… I take multiple medications due to uncontrolled blood sugars and it becomes quite depressive.”* | [General views/perception of FLO]: *“FLO is a good system; the messages are informative and educational. I also find the reminders convenient. However, my English is just okay, it’s not the best I’m lucky I can read the messages, but I think there should be translated content instead, to help others who don’t read English at all.”*  [Coping, Religious views/adoption to FLO]: *“The disease is from Allah, we have all trust in Allah, only He can cure us, even advances in technology (mobile phones or text messages) don’t have that power.”* | [General views and perception of FLO]: *“FLO messages have been just great. I think it is very quick and easy to use. They keep me well informed with new updates and encourage me to keep a record of my self-management progress.”*  [Coping, Religious views/ adoption to FLO]: *“I have been very depressed since the diagnosis. My religion and faith in God have helped me feel more normal. FLO has also helped me bounce back with my life.”* |
| Level 3 - Following and understanding prescribed regime, positive outlook on Diabetes. Good activation level | Level 1 - Poor self-management, non-adherent to medications. Low activation level | Level 2 - Non-adherent sometimes due to forgetfulness. Lower activation level | Level 1 - Adherent with some messages and self-management tasks. Low patient activation levels | Level 4 - Adherent with messages and self-management regime. High patient activation levels |
| *“Well, scriptures do say, God can do anything, he can cure you if he wants, I do my prayers every day, so God gives me a reason to cope.”* | *“They tried to persuade me, they kept telling me the complications, but I didn’t care at that point, I thought if it is meant to be it will be. It is all in God’s hands. It’s the same with FLO, I don’t think it’s any good.”*  *“I do believe whatever happens is for a reason. I am religious and believe in God. I am a firm believer and I look for guidance in God. God can do anything; he can cure me if he wanted to.”* | *“I am Hindu, but I don’t really follow my religion. I don’t think God or religion has anything to do with my condition. My condition is to do with my own doing. diabetes is due to biological reasons not because of religion. I do think science and technology are quite common and important these days.”*  *“I am not really that religious, I do believe in God… But in terms of my health, I am more reliant on scientific evidence and treatments that can treat my symptoms.”* | *“Faith and self-management are two sperate factors. Using FLO does not intervene with my religion.”*  *“Although, technology is good and the texts are educating me, it doesn’t matter how much you are suffering, only God can restore happiness and health.”* | *“I attend the temple daily, praying to God and seeing people from the community has provided a lot of emotional support for me.”*  *“When I first heard the diagnosis of diabetes, I was really down, and when I was on the ward it was all old people, I was like where are all the young people gone, is this a disease that only old people get? I just wanted to hide myself, what is happening to me? My healthcare providers have really supported me and introduced me to FLO which has helped me even more.”*  *“I think the first most important thing is to be educated, only then you can understand your illness properly. I think second to that would be to have the motivation and courage to want to change. “* |

## **Appendix 10: Table 9.** *Self-Efficacy of SA Users and Non-Users*

| **U11 Context** - *Enhanced self-efficacy*  Male (48), Pakistani, college education, employed. Diabetic and hypertensive, diagnosed with CKD stage three last year. Reported feeling depressed and hopeless. Introduced to the FLO system by the diabetes Endocrinologist. No issues with technology or IT, so decided to sign up to FLO, receives daily messages to support self-management. | **NU10 Context -** *Low self-efficacy*  Male (55), Indian, secondary school education in India, employed. Diabetic and hypertensive. Punjabi speaker, does not speak English well. Expressed the importance of food in the Indian culture and difficulty to change this, even to improve self-management and using the FLO. |
| --- | --- |
| **FLO User** | **FLO Non-User** |
| [General views/perception of FLO] “*Messages are very useful and helpful. Has helped me connect with my care providers when I need support. The information provided in texts helps me do better.”*  [Self-efficacy and adoption to FLO]: *“FLO has given me hope, and has made me want to do better for myself and my family. I don’t think I can manage as effectively without it. Since I have started using it, I have developed a better routine. I make sure I am checking my vitals, exercising often and eating right.”* | [General views/perception of FLO]: *“I don’t really think I would follow the messages as I am not good with technology… I can speak to the doctor face to face when I need to.”*  [Self-efficacy and adoption to FLO]: *“I do not want to change my lifestyle. Therefore, I don’t want to start using the system now. I cannot change what I have been doing my whole life” [referring to lifestyle].* |
| Increase in self-efficacy, and high activation levels (level 4) (p=0.00) | Low self-efficacy, and low activation levels (p=0.00) (level 1) |
| *“When I was faced with a diagnosis of a long-term illness such as diabetes and CKD, it came as a shock to me. I did not know what to do, I mean… the help was there but I thought ‘what’s the point’. I did not feel like trying anymore. But once I was introduced to FLO, my life changed.”*  *“I am always improving now according to my annual appointments and that is the way forward, all thanks to FLO.”*  *“Although I have FLO, my family have been my main supporters without them, this journey would be impossible.”* | *“I like my current lifestyle, to eat things like curry and samosas, pakoras, biryani is our food. It is our identity. I don’t think me or my family will ever change that. We don’t eat fried food as often anyway so it is okay.”*  *“I don’t think a text messaging system will make me change my ways. I guess they can offer good advice but for me that won’t work, I don’t think I will follow it. For that reason, I never signed up.”* |
